# English-Learning Infants’ Developing Sound System Guides Their Early Word Learning

**DOI:** 10.3390/bs15050605

**Published:** 2025-05-01

**Authors:** Suzanne Curtin, Susan A. Graham

**Affiliations:** 1 Department of Child and Youth Studies, Brock University, St. Catharines, ON L2S 3A1, Canada; 2 Department of Psychology, University of Calgary, Calgary, AB T2N 1N4, Canada; grahams@ucalgary.ca

**Keywords:** phonological development, distributional learning, word learning

## Abstract

Children appear to acquire new words effortlessly from complex auditory input. However, this process is highly intricate, requiring the simultaneous integration of phonetic and phonemic details, prosodic cues, and grammatical structures. Furthermore, different components of a language’s sound system—such as phonemes, syllables, and prosodic features—appear with different frequencies in the input and follow distinct patterns of distribution in speech. This article reviews research that illustrates how infants’ growing understanding of their native language sound system facilitates their acquisition of new words.

## 1. Introduction

Language is a critical achievement, emerging early in development and setting the stage for numerous later accomplishments, including literacy skills, academic achievement, social competence, and psychosocial adjustment (Conti-Ramsden, 2008; Dubois et al., 2020; Hentges et al., 2021; Le et al., 2021; Norbury et al., 2016; Preston et al., 2010; Wadman et al., 2008; Wieczorek et al., 2025). Although it can appear effortless for many children, the acquisition of language is an extraordinarily complex process that requires the seamless integration of multiple cognitive, social, and perceptual skills. Consider, for example, the steps involved in learning a new word. To learn a new word, an infant must establish some type of mapping between a sound pattern and a referent in an environment that is both linguistically and conceptually noisy. That is, in a busy auditory and visual environment, infants must isolate the phonological structure of a word, identify and link it to its referent, store this connection, and later recognize or recall the word–referent association. In addition, children must also contend with phonetic/phonological variation in the words they hear. Despite these challenges, infants navigate the word-learning process quickly, progressing from a vocabulary of roughly six words at 12 months to over 50 words by 18 months (Frank et al., 2017). This rapid rate of lexical acquisition raises the possibility that the system for word learning must be constrained in some way (see Curtin & Zamuner, 2014; Graham et al., 2016; Westerman & Mani, 2018 for broad reviews of word learning). In this study, we review research examining one candidate constraint that may narrow the search space when learning new labels: infants’ knowledge of their native language sound system. We review how infants’ knowledge of sound forms constrains mappings, how experience with sound patterns influences early word–object mappings, and how the stored knowledge of object labels can influence mappings of other grammatical forms to their referents.

## 2. The Phonological System and Early Word Learning

In this section, we review research demonstrating how infants’ growing phonological understanding influences their learning of object words. We focus largely on studies with English-learning infants that examine one of the initial stages of word learning: namely, connecting a sound form to an object. These studies typically draw upon the Switch task (Werker et al., 1998), a word–object association task devoid of contextual or referential cues. In this paradigm, infants (and their caregivers) are seated in front of a monitor with a speaker below it. First, infants are presented with a pre-test trial consisting of a visual stimulus paired with an auditory stimulus (usually a sound form). The infant is then presented with blocks of habituation trials; in each trial, a novel visual stimulus is presented on the screen moving back and forth while an auditory stimulus is played. Once the infant habituates to a preset criterion of change in looking time from the first block of trials (e.g., 65%), the two test trials automatically begin. During the *same test trial*, the object–auditory form pairing given during habituation is presented. During the *switch test trial*, a mismatch (switch) in the object–auditory form pairing is presented. At the end, a post-test trial using the same stimulus pairing as in the pre-test is presented. If infants have learnt the sound–form object mapping, they should look at the *switch* trial for longer than the *same* trial. Studies have shown that infants as young as 12 to 14 months can associate novel words with objects in this paradigm (Curtin, 2009; Tsui et al., 2019; Werker et al., 1998).

Given that infants are provided with no referential or intentional cues from a speaker, the use of the Switch task allows researchers to examine infants’ preferences for different types of sound forms in a purely associative word learning task. Using this paradigm, research has examined the kinds of sound forms that infants consider as potential labels during the early phases of word learning, including: (1) the influence of the phonological structure of the sound form; (2) the influence of the phonotactics of the word form. Much of this research has focused on infants during the early stages of word learning, around 12 months of age.

### 2.1. Infants’ Attention to the Phonological Structure of the Sound Form

Studies in this line of research have explored whether infants prioritize certain types of sound sequences, specifically those resembling typical content words, over other linguistic sounds when learning new labels. For example, H. MacKenzie et al. (2011) contrasted 12-month-olds’ sound–object mappings when presented with three types of sound forms that varied in their similarity to typical content words in English: (1) novel CVC words that consisted of three phonologically legal segments in English (e.g., fep); (2) communicative sounds that share some characteristics with words, such as the ability to convey meaning in context (e.g., ooh shh); and (3) single consonantal sounds that share structural similarities with communicative sounds but lack any possible meaning (i.e., /l/). The results indicated that English-learning 12-month-old infants successfully linked objects with well-structured CVC words but did not link objects with either communicative sounds or isolated speech sounds.

By the end of their first year, infants’ associative learning mechanisms become sensitive to differences in sound forms (i.e., differences between communicative sounds and CVC words). As a result, they associate only words with meaningful linguistic content. These findings suggest that simple sound–object associative pairings are not enough to form mappings. Instead, infants need well-structured, noun-like words to co-occur with objects for such associations to develop. In the next section, we review evidence that this sensitivity includes attention to native-language phonotactic sequences.

### 2.2. Sensitivity to Phonotactic Sequences in Word–Object Associations

Phonotactics are the constraints within a specific language that determine which combinations of sounds are permissible and how they can be sequenced. For example, in English, the combination “str” is allowed at the beginning of a word (as in “street”), but “tsr” is not. By nine months, infants can distinguish between legal and illegal sound combinations (Jusczyk et al., 1993). They can also identify the speech sound combinations and positions that are more likely (Jusczyk et al., 1994) and show a preference for legal sound combinations in appropriate positions (Friederici & Wessels, 1993). This sensitivity to phonotactic probabilities includes sound positions both within and across word boundaries (Mattys et al., 1999). Thus, by this age, infants have developed an understanding of legal sound combinations and positional constraints at the beginnings and ends of words in their native language (see Sundara et al., 2022 for a recent meta-analysis).

This understanding of legal sound combinations emerges through experience with the native language. While infants of six months can discriminate between stop–liquid clusters at the beginning of words (e.g., gl, bl, dl), they do not demonstrate a preference for native-language sound sequences until around nine months of age (Archer & Curtin, 2011). This is likely due to their experiences with these clusters in the input. For example, nine-month-old infants prefer listening to stop–liquid clusters with high type frequency rather than low or zero (non-native) type frequency (Archer & Curtin, 2011). Sensitivity to the type frequency of onset clusters is further demonstrated in word segmentation tasks that require infants to recognize words embedded in passages of continuous speech, where nine-month-old infants use both phonotactic legality and statistical frequency to segment words from fluent speech. That is, they are more successful at finding words when the beginnings of those words are both legal and frequent in their language environment (Archer & Curtin, 2016). Interestingly, even when a sound combination is infrequent, infants can still learn to use it for word segmentation if they have some prior experience with it. The key is that the sound combination must be possible in the language (even if it is rare). That is, additional exposure to impossible sound combinations in the onset position of a syllable (“dl-”) does not seem to help with segmentation (Archer et al., 2021) suggesting that pre-familiarizing infants to illegal clusters is not sufficient to support use of that information. Together, these findings highlight the importance of overall experience with the language in shaping infants’ developing phonotactic knowledge.

Drawing upon this body of research, studies have examined whether this phonotactic sensitivity governs infants’ word–object associations. Using the Switch task, research has demonstrated that infants’ early object–word associations reflect their growing understanding of legal phonotactic sequences in their language. For example, 12-month-old English-learning infants reject labels with illegal onset sequences, such as the Czech form *ptak* (H. MacKenzie et al., 2012; H. K. MacKenzie et al., 2014). However, 12- and 17-month-old infants do accept atypical forms, such as the Japanese form *sika*, which contains allowable phonemes and is phonotactically legal in English but is differs in how it is phonetically realized in English, as object labels (H. MacKenzie et al., 2012; San Juan et al., 2017). This willingness to accept forms with legal sound patterns but phonetic irregularities suggests that infants tolerate some phonetic variation, likely due to variability in their language input. However, they consistently reject forms that violate their native language’s phonotactic rules. Similarly, Graf Estes et al. (2011) found that 18-month-old infants mapped phototactically legal word forms (e.g., *dref*, *sloob*) to objects but not phototactically illegal word forms (*dlef*, *sroob*) in the looking-while-listening task.

In sum, infants in the early stages of learning to produce words exhibit preferences for specific types of word forms as object labels. We propose that these preferences support infants’ language development by narrowing their search for suitable word forms in an acoustically busy environment. Next, we consider whether any experience with word forms supports word–object mapping or whether infants have sophisticated knowledge of word categories that might impact learning.

### 2.3. Mapping of Less Probable Object Labels to Referents

Infants’ language exposure extends beyond just nouns, as the linguistic environment is diverse and incorporates a variety of grammatical forms. During early mappings, content words such as nouns are predominant, but exposure to other forms, such as the small set of highly frequent function words (e.g., articles and prepositions) also takes place. 

While all types of words are represented in the input, there are acoustic and distributional characteristics that distinguish between these word types. Content words are typically longer and carry stress in the sentence. Function words tend to have simpler syllable structures (e.g., CV, VC, or V) and frequently feature reduced vowels such as the schwa (/ə/), in contrast to content words. These distinctions are not unique to English but are found across various languages, including Dutch, French (Monaghan et al., 2007), Mandarin, and Turkish (Shi et al., 1998). Another important difference lies in their frequency: function words appear more often but with fewer unique types, while content words are more varied but individually occur less frequently in speech (Shi et al., 2006; Shi & Lepage, 2008). Sensitivity to the differences between content and function words emerges early in life, as indicated by six-month-old infants’ preferences for listening to content words over function words (Shi & Werker, 2001).

Research has demonstrated that infants can recruit their sensitivity to the differences between content and function words to guide their acceptance of novel object labels (Geffen et al., 2023; H. M. MacKenzie et al., 2012; H. K. MacKenzie et al., 2014). That is, 12-month-old English-learning infants will successfully map content-like words (e.g., *fep*) to objects but not function-like words (i.e., words with CV or VC structures and lax vowels; e.g., /kʌ/ and /ɪv/; H. M. MacKenzie et al., 2012; H. K. MacKenzie et al., 2014). The finding that infants will not map function-like words to objects was recently replicated in a series of studies conducted by Geffen et al. (2023) who demonstrated that 12-month-old infants will not map function-like words to objects, even when these words are presented in a sentential context that suggests they are a content word (i.e., “look at the /kʌ/”) or when the vowels are elongated, more in keeping with CV and VC content words (e.g., *koo* /ku/ and *ook* /uk/, similar to words such as *shoe* and *oak*).

Content words are not limited to nouns: they also include verbs, adjectives and adverbs. In the case of English content words with more than one syllable (e.g., CVCV), 94% of disyllabic nouns demonstrate a strong–weak stress pattern (CVcv), known as trochaic, and 69% of disyllabic verbs exhibit a weak–strong stress pattern (cvCV), known as iambic (Kelly & Bock, 1988). Infants start using stress information to segment the speech stream at around seven months of age and demonstrate a trochaic preference (Curtin et al., 2005; Johnson & Jusczyk, 2001; Jusczyk et al., 1999; Polka et al., 2002; Thiessen & Saffran, 2007). English-learning infants can segment consonant–initial iambic verbs and vowel-initial iambic verbs by the time they reach 16.5 months of age (Nazzi et al., 2005). At 12 months of age, infants can distinguish two trisyllabic object labels that differ minimally only in their stress pattern (i.e., *BEdoka*, *beDOka*; Curtin, 2009) and will detect a mismatch if the segments stay the same but the stress is shifted to another syllable (Curtin, 2010, 2011). These findings suggest that, while infants are sensitive to stress information, they have yet to learn that it is not a productive feature of English. That is, very few words in English are distinguished primarily by which syllable is stressed (e.g., *REcord* (noun) versus *reCORD* (verb)), and they typically fall into different grammatical categories. Taken together, these studies demonstrate that infants have knowledge of the predominant stress pattern of their language and use this to find words in the speech stream. Furthermore, they have rich representations of word forms that encode the stress pattern, but they do not fully understand the role of stress in disambiguating words. The question is, then, when they begin to know that English nouns tend to be trochaic and verbs iambic, and that word stress is a non-productive feature of the language (i.e., altering the stress of a word does not typically signal a change in word meaning).

To explore whether infants know that iambic stress is typically associated with verbs in English, Curtin et al., 2012 tasked 16-month-old infants with listening to two novel words with either verb-like iambic stress or noun-like trochaic stress, such as *tiDU* /ti’du/ or *KOme* /’kome/ respectively. Each word was paired with a single novel object performing one of two path actions, a plus (+) path motion and a circle motion. Only infants in the iambic stress condition learned the association between the novel words and the path actions, suggesting that English-learning infants have developed a bias to expect disyllabic action labels to have iambic stress patterns, consistent with native language stress patterns (Curtin et al., 2012).

In a subsequent study, 17-month-old infants were tested to see whether they would use word stress to disambiguate a labeling event involving objects and actions with equal plausibility (Campbell et al., 2018). That is, would infants map a wordform such as /’kome/ that has a typical noun stress pattern in English, to an object and/or a path action, and would they do the same for a wordform that has a typical verb stress pattern, such as /ti’du/. Infants were familiarized with two words paired with two objects, each associated with a distinct path action. Following habituation, the infants underwent testing involving either a change in object but not path action, or a change in path action but not the object. Infants exposed to verb-friendly iambic labels exhibited increased looking times when both the action and the object switched, indicating a sensitivity to the label’s stress pattern. In contrast, infants taught with noun-friendly trochaic labels showed heightened looking times only when the object switched, suggesting a different mapping of stress patterns to objects and actions in ambiguous labeling events. In other words, infants associate iambic labels with both actions and objects, while trochaic labels are primarily linked to objects rather than actions, suggesting that 17-month-old infants are capable of using trochaic lexical stress to navigate word learning in ambiguous contexts, but the influence of iambic stress cues on infants’ mappings of actions or objects may not be preferential. Thus, experience constrains which mappings are made with trochaic labels, but infants remain unclear on the role of iambic stress.

These research findings raise the question of whether infants are aware that word stress is not an effective way to distinguish between words in English. To address this question, 17-month-old infants were presented with pairs of familiar objects (e.g., a picture of a baby) while a target label was pronounced either correctly with the appropriate stress, such as *BAby* /’beɪbi/, or incorrectly with a mis-stressed pattern, like *baBY* /beɪ’bi/ (Campbell et al., 2019). Infants successfully associated both the correctly stressed and mis-stressed labels with the target objects, but they took longer to focus on the target when hearing the mis-stressed label. Further investigation assessed whether infants recognize the non-productive role of stress in English by exposing them to a familiar object paired with a novel object while presenting them with correctly stressed or mis-stressed familiar words. The outcome indicated that infants linked the correctly stressed label to the familiar object but did not consistently associate the mis-stressed label with either the target or distractor objects. These findings suggest that word stress influences the processing of familiar words and indicate that infants of this age are developing an awareness that altering the stress pattern of a familiar word does not consistently indicate a new referent.

### 2.4. Constraints vs. Preferences

The research reviewed above demonstrates that 12- to-18-month-old infants show robust tendencies to privilege particular word forms as labels for objects and have a burgeoning knowledge of how word stress aligns with different types of words. As we noted earlier, these tendencies assist in word learning by narrowing the search space for candidate labels, allowing infants to focus on establishing word-objects mappings. It remains to be determined, however, whether these tendencies reflect constraints on learning versus preferences for particular word forms. A number of studies have addressed this question by examining whether infants show flexibility in accepting different types of word forms if presented with social-referential cues.

Recall that studies documenting infants’ preferences for word forms have drawn heavily on the Switch task, a purely associative word learning task. To examine whether the addition of referential cues shifts infants’ preferences, researchers have adapted the classic Switch task (Werker et al., 1998), incorporating a referential training component (e.g., Fennell & Waxman, 2010). In these studies, infants are tested in one of two conditions, each designed to shape their perception of object labels in different ways. In the Name Training condition, infants are first introduced to familiar objects, each paired with its conventional English noun (e.g., dog), followed by word–object pairings as in the traditional Switch task. This training is intended to provide a strong cue that words presented in this task can serve as meaningful labels. In the Exclaim Training condition, familiar objects are paired with common exclamations (e.g., *Oh!*), instead of nouns. This condition acts as a comparison, allowing researchers to explore how infants respond when the referential nature of words remains uncertain.

Studies have shown that providing referential cues in the Modified Switch task can lead infants to adjust their preferences for particular word forms. That is, when given cues directing their attention toward object labels, English-learning infants will associate phonotactically illegal word forms (e.g., *ptak*) or non-native speech sounds (e.g., [!a]) with objects (H. K. MacKenzie et al., 2014; May & Werker, 2014; Vukatana et al., 2016). However, this flexibility has limits. Even with additional referential information, infants reject isolated consonantal sounds (e.g., /l/) or function-like words (e.g., /kʌ/ and /ɪv/) as object labels. This suggests that structurally simple forms (i.e., C, CV, or VC structures) are not perceived as valid object labels (H. K. MacKenzie et al., 2014).

As infants approach their second birthday, their ability to flexibly map forms to objects becomes more restricted (Graham & Kilbreath, 2007; Namy & Waxman, 1998; Suanda & Namy, 2013; Woodward & Hoyne, 1999). However, the trajectory of this restriction varies depending on the type of form being mapped. By 17 to 20 months, infants no longer associate objects with words that have phonotactically illegal onsets (Graf Estes et al., 2011; Vukatana et al., 2016) or non-native speech sounds (May & Werker, 2014). Despite this, they continue to associate symbolic gestures with objects (Graham & Kilbreath, 2007; Namy, 2001; Suanda & Namy, 2013). Between 22 and 26 months, this flexibility further diminishes, and infants stop mapping gestures to objects altogether (Graham & Kilbreath, 2007; Namy & Waxman, 1998). For example, Namy and Waxman (1998) introduced 18- and 26-month-old infants to object categories (e.g., fruit, vehicles) using either a novel word or a symbolic gesture. Both age groups interpreted novel words as category labels. However, gesture interpretation changed with age: 18-month-old infants treated gestures like words, while 26-month-old infants did not. We note that, under specific conditions, infants older than 24 months may still display some flexibility, associating nonlinguistic sounds (Henderson et al., 2015) and gestures with objects (Namy et al., 2004). For example, with additional training with gestures, the 26-month-olds infants in Namy and Waxman’s (1998) study linked gestures with objects.

## 3. Conclusions

In sum, over the course of the first two years of life, infants track and store information about their native language sound system and use this information to learn which sequences of segments are legal in their language, which types of sound patterns are typically associated with objects, and what prosodic information tends to align with nouns. Infants are highly skilled learners, and they tend to restrict their mapping of forms that are unfamiliar to them when lacking additional information, whether it is referential or related to the task at hand. These findings indicate that infants’ language acquisition is influenced and directed by their experiences, the context in which they are learning, the input they receive, and the current state of their linguistic abilities (Werker & Curtin, 2005).
